# A Detection Method for Seeding Temperature in Czochralski Silicon Crystal Growth Based on Multi-Sensor Data Fusion

**DOI:** 10.3390/s26020516

**Published:** 2026-01-13

**Authors:** Lei Jiang, Tongda Chang, Ding Liu

**Affiliations:** 1 School of Automation and Information Engineering, Xi’an University of Technology, Xi’an 710048, China; 2 Crystal Growth Equipment and System Integration National & Local Joint Engineering Research Center, Xi’an University of Technology, Xi’an 710048, China; 2230320147@stu.xaut.edu.cn

**Keywords:** Czochralski silicon single crystal, seeding temperature detection, deep learning, multimodal fusion, regression model, wavelet transform

## Abstract

The Czochralski method is the dominant technique for producing power-electronics-grade silicon crystals. At the beginning of the seeding stage, an excessively high (or low) temperature at the solid–liquid interface can cause the time required for the seed to reach the specified length to be too long (or too short). However, the time taken for the seed to reach a specified length is strictly controlled in semiconductor crystal growth to ensure that the initial temperature is appropriate. An inappropriate initial temperature can adversely affect crystal quality and production yield. Accurately evaluating whether the current temperature is appropriate for seeding is therefore essential. However, the temperature at the solid–liquid interface cannot be directly measured, and the current manual evaluation method mainly relies on a visual inspection of the meniscus. Previous methods for detecting this temperature classified image features, lacking a quantitative assessment of the temperature. To address this challenge, this study proposed using the duration of the seeding stage as the target variable for evaluating the temperature and developed an improved multimodal fusion regression network. Temperature signals collected from a central pyrometer and an auxiliary pyrometer were transformed into time–frequency representations via wavelet transform. Features extracted from the time–frequency diagrams, together with meniscus features, were fused through a two-level mechanism with multimodal feature fusion (MFF) and channel attention (CA), followed by masking using spatial attention (SA). The fused features were then input into a random vector functional link network (RVFLN) to predict the seeding duration, thereby establishing an indirect relationship between multi-sensor data and the seeding temperature achieving a quantification of the temperature that could not be directly measured. Transfer comparison experiments conducted on our dataset verified the effectiveness of the feature extraction strategy and demonstrated the superior detection performance of the proposed model.

## 1. Introduction

Monocrystalline silicon is a fundamental semiconductor material widely used in the photovoltaic and semiconductor industries [1]. Currently, the Czochralski (CZ) method is the leading technique for the large-scale industrial production of monocrystalline silicon [2]. In the CZ process, polysilicon feedstock is melted in a crucible, after which a seed crystal is brought into contact with the molten surface. By controlling crystal rotation and slowly pulling the seed upwards, the silicon melt solidifies epitaxially along the crystallographic orientation of the seed. Through successive stages including seeding, shouldering, body growth, and tail growth [3], a monocrystalline silicon ingot is ultimately formed. Specifically, the seeding stage marks the beginning of crystal growth. When the seed crystal is dipped into the melt, the contact interface reaches a transient thermal equilibrium, and the melt adheres to the periphery of the seed due to surface tension. As the seed is lifted, a supercooled region develops above the melt–seed interface [4], enabling silicon atoms to attach and crystallize along the seed lattice direction. The thermal state during this process is highly sensitive. Excessively high temperatures may slow or even inhibit crystallization, while insufficient temperatures may cause abnormal neck thickening or even lateral crystallization.

Stable crystal growth requires well-controlled temperatures at the solid–liquid interface. However, this interface lies within the melt and cannot be directly measured. Its temperature state can only be inferred from surrounding measurable parameters. During the stable stage prior to seeding, the growth process must be guided into an appropriate temperature range [5]. At present, in industrial production, the judgment of seeding temperature relies heavily on manual experience, particularly through the visual assessment of the bright ring at the contact interface. Moreover, the time taken for the seed crystal to reach the specified length is strictly controlled (called the seeding duration) to ensure the consistency of the solid–liquid interface temperature at the moment of seeding.

Only a limited number of studies to date have investigated crystal behavior during the seeding stage, and most existing work has concentrated on growth mechanisms or thermal field modeling. Wang Zhengsheng et al. examined the relationship between the crystal growth rate at the meniscus and the thermal field, as well as the meniscus morphology [6]. Halima Zahra Bukhari et al. applied ray tracing to a physical model of the solid–liquid interface to simulate the bright ring on the meniscus, examined its relationship with the crystal rod state [7], and subsequently conducted diameter detection based on the optical features of the bright ring. Shiori Ueta et al. extracted growth ridges from bright ring images using a difference-of-Gaussian approach [8]. Regarding methods for temperature detection during the seeding stage, Zhao Yue et al. utilized meniscus image information and employed a least squares support vector machine to divide the seeding temperature into five intervals through multiple binary classification decisions [9], achieving a certain degree of temperature detection. The existing methods rely solely on input signals from meniscus images for geometric feature extraction, utilizing similar information for manual qualitative judgment. The limited geometric feature information in this method cannot fully reflect the crystal growth temperature, and it has not yet fully utilized the available multi-sensor data in the rod pulling furnace. Moreover, temperature detection is still limited to qualitative interval classification, and there is still research space for more refined quantitative detection. Techniques incorporating multi-sensor data and introducing posterior information have the potential to achieve more accurate and refined seeding temperature detection.

When processing one-dimensional signals, analysis can be performed in the time domain, frequency domain, or time–frequency domain. Time-domain analysis directly examines variations in the data sequence over time [10], with statistical descriptors such as mean, variance, skewness, and kurtosis characterizing the distribution of the signal. In addition, autocorrelation functions and linear prediction coefficients can reveal internal correlations and structural properties of the signal. Frequency-domain analysis converts the signal from the time axis to the frequency axis via Fourier transform, thereby disclosing inherent frequency components and energy distribution. This approach enables the clear identification of periodic or harmonic components that are difficult to observe directly in the time domain [11]. In the time–frequency domain, the wavelet transform provides a joint representation of temporal and spectral characteristics [12,13], capable of precisely capturing time-varying features that cannot be adequately revealed via the Fourier transform [14]. For image-based feature extraction, convolutional neural networks (CNNs) [15] remain the mainstream method. Numerous network architectures have been developed based on CNNs, including VGGNet [16], ResNet [17], and the Swin Transformer [18]. Multimodal fusion leverages complementary information from different sources to achieve more comprehensive and accurate processing and decision-making. It is generally categorized into data-level fusion, feature-level fusion, and decision-level fusion [19]. Data-level fusion directly integrates raw data from multiple modalities [20]; feature-level fusion combines features extracted from each modality [21]; and decision-level fusion aggregates decision outcomes from multiple modalities to produce the final result [22].

To address the limitations of existing seeding temperature detection methods, we introduced data from multiple sensors on the Czochralski furnace equipment and quantified the immeasurable temperature by using the seeding duration as the target variable for evaluating the seeding temperature. Specifically, we propose an improved multimodal fusion regression network, termed the SA-CA-MFF-RVFLN model. Meniscus image data and one-dimensional signals collected from the central and auxiliary pyrometers are used as multimodal inputs. The one-dimensional signals are transformed into time–frequency representations via wavelet transform, from which both time–frequency domain features and meniscus features are extracted. Following a two-level multimodal feature fusion (MFF) and channel attention (CA) mechanism, together with spatial attention (SA) masking, the fused features are fed into a random vector functional link network (RVFLN) to establish regression mapping between the input features and the seeding duration. In this way, an indirect relationship between multi-sensor data and the seeding temperature state is constructed.

The main contributions of this work are as follows.

A novel temperature detection approach is proposed, in which the duration of the seeding stage is used as an indicator of the initial seeding temperature conditions. This duration serves as the regression target, enabling the prediction of the seeding duration from multi-sensor data and thus facilitating indirect seeding temperature assessment.

A multimodal fusion regression network, SA–CA–MFFRN, is developed. Through a two-level fusion structure incorporating MFF and CA modules, features from different sensors are effectively integrated. Samples are labeled with the measured seeding duration. After spatial masking through the SA module, the fused features are regressed using a multi-layer perceptron (MLP) to measure the quantified seeding temperature.

An enhanced architecture, SA-CA-MFF-RVFLN, is further designed by integrating the trained feature extraction network into an RVFLN via transfer learning. This integration preserves model accuracy while reducing training costs and mitigating overfitting risks. The transfer learning results also validate the effectiveness of the extracted features, providing support for the model’s generalizability under varying practical production scenarios.

## 2. Data Collection

**Figure 1:**
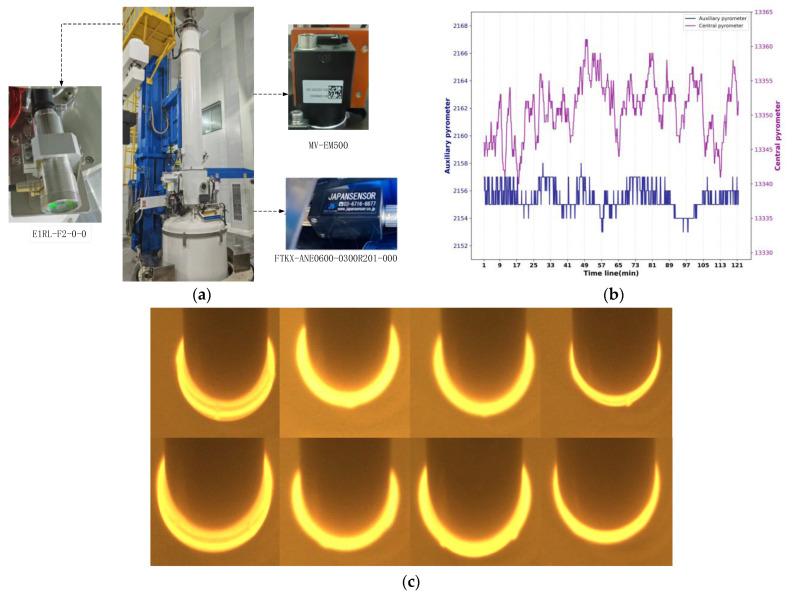
Data collection: (**a**) the layout of on-site equipment and sensors; (**b**) the sample collected using the central pyrometer and auxiliary pyrometer; (**c**) different meniscus shape images collected using the camera at different temperatures.

The experimental data used in this study were obtained from a 12-inch Czochralski furnace. This system is capable of producing CZ silicon single crystals with diameters ranging from 100 to 308 mm. The quartz crucible has a diameter of 800 mm, and the maximum polysilicon loading capacity is 450 kg. The maximum output power of the side heater is 180 kW, while that of the bottom heater is 80 kW. The rotational speed ranges are 0–15 rpm for the crucible and 0–20 rpm for the crystal. The adjustable ranges of the crucible lifting rate and the crystal lifting rate are 0–1.3 mm/min and 0–6 mm/min, respectively. The maximum attainable vacuum level is 0.3 Pa. Regarding the sensing devices, meniscus image acquisition was performed using an MV-EM500 camera manufactured by Microvision, Beijing, China. The central pyrometer mounted above the melt was an E1RL-F2-0-0 infrared sensor produced by Fluke, Everett, WA, USA. The auxiliary pyrometer installed on the crucible sidewall was an FTKX-ANE0600-0300R201-000 infrared sensor manufactured by JAPANSENSOR CORPORATION, Tokyo, Japan. Regarding the data acquisition section, the single crystal silicon ingot prepared in the experiment had a diameter of 308 mm, a seeding length of 300 mm, and a constant diameter length of 1200 mm. In the experimental stage in this study, the gas flow rate was 90 L/min, the furnace pressure was 20 Torr, the crystal rotation rate was 10 rpm, the crucible rotation rate was 0.5 rpm, the magnetic field intensity was 1000 Gauss, with the magnetic field positioned 60 mm below the liquid surface, and the melt gap was 50 mm. The image sensor sampled at 10 frames per second, and the sampling frequency of both the central pyrometer and the auxiliary pyrometer was once per second. The labeling of the classification dataset was completed by laboratory engineers based on experience, as shown in Figure 1.

Through multiple crystal pulling experiments, a total of 3762 sets of sensor data were collected at different moments of the melt–seed contact state, together with the corresponding meniscus images. Due to the existence of gaps between the heater and the crucible and the time required for heat conduction within the melt [23], there is a lag between heater power changes and their manifestation at the solid–liquid interface. Based on accumulated experimental experience, this lag is approximately 60 min. Therefore, each meniscus image was matched with sensor data from the preceding 60 min period, corresponding to 720 data points. The dataset was divided into training, validation, and test sets in a 7:1:2 ratio. Input images were normalized to a resolution of 256 × 256. 

To more effectively utilize the collected one-dimensional signals and improve the accuracy of temperature state detection, the one-dimensional data were processed using the continuous wavelet transform (CWT). The wavelet transform is defined as follows:

(1) $$CWT_{a,b}(t)=\frac{1}{\sqrt{a}}\int_{-\infty }^{+\infty }f(t)\cdot \varphi^{*}(\frac{t-b}{a})dt$$

Here, $f\left(t\right)$ denotes the original function, and $\varphi \left(t\right)$ represents the wavelet basis function.

According to the principles of Czochralski silicon single crystal growth, lower temperatures lead to faster crystallization, whereas higher temperatures slow the crystallization rate. Moreover, the system remains in a stable state for a relatively long period prior to seeding, and the thermal field inside the furnace can be regarded as quasi-steady throughout the seeding process. Therefore, the thermal characteristics during both the pre-seeding stage and the seeding initiation stage are correlated with the final seeding duration (the seeding length for all batches in this study was fixed at 300 mm). This posterior knowledge supports the use of the seeding duration as an indirect indicator of the seeding temperature. Based on this, the one-dimensional signals collected before seeding were temporally aligned with the corresponding meniscus images, while the seeding duration was used as the regression label. In this manner, a regression dataset was constructed. The post hoc initialization times of these samples ranged from 68 to 95 min. 

## 3. Proposed Method

**Figure 2:**
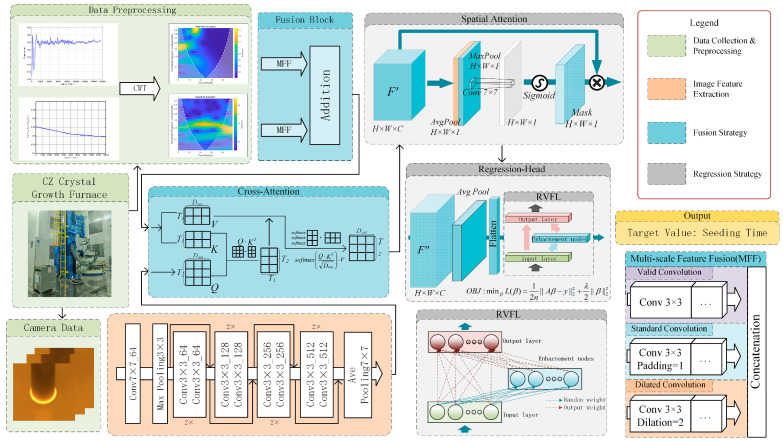
SA-CA-MFF-RVFL network structure.

To accurately evaluate the seeding temperature state, in this paper, we present a multimodal fusion regression network, termed SA-CA-MFF-RVFLN, the overall framework of which is illustrated in Figure 2.

During monocrystalline silicon growth, the one-dimensional signals were first converted into time–frequency representations using the continuous wavelet transform. For time–frequency domain feature extraction, a multi-scale feature fusion module was designed to integrate features from the original, down-sampled, and dilated convolution scales. For the meniscus images captured using the industrial camera, a ResNet-based feature extraction module was employed. After feature extraction, a CA module was used to fuse heterogeneous sensor information, with the time–frequency domain features acting as the Key/Value and the meniscus image features serving as the Query, enabling cross-modal feature integration. The fused features were subsequently processed via an SA mechanism for feature masking and finally fed into the RVFLN for the regression of the seeding duration, to detect the seeding temperature.

### 3.1. Image Feature Extraction Module

Since manual evaluation of the seeding temperature primarily relies on visual inspection of the bright ring on the meniscus, in this study, we adopted the meniscus image as input and built a ResNet-based feature extraction network for classification and regression experiments. The structure of this part corresponds to the region of the same name in Figure 2 (see legend).

ResNet, proposed by Kaiming He et al. (2016) [17], addresses the issues of vanishing gradients and performance degradation in deep neural networks. Its core innovation lies in the residual learning structure, which consists of a feedforward network combined with skip connections.

Instead of directly learning the underlying mapping of $x\to H\left(x\right)$, the residual network learns the difference between the two, that is, the residual. Thus, the forward propagation becomes:

(2) H(x)=F(x)+x

By learning $F\left(x\right)+x$ rather than directly fitting $H\left(x\right)$, the network optimization is simplified. This allows the model to deepen effectively, thereby enhancing the final classification accuracy.

### 3.2. Fusion Strategy

As mentioned earlier, meniscus features constitute an important indicator in determining the current temperature state, with the data obtained from the central and auxiliary pyrometers capturing the thermal dynamics in other regions near the melt. To effectively integrate and exploit the complementary information provided by these sensors, for this study, we designed a two-level multi-scale fusion strategy to accommodate the heterogeneous characteristics of multimodal sensor inputs.

**Figure 3:**
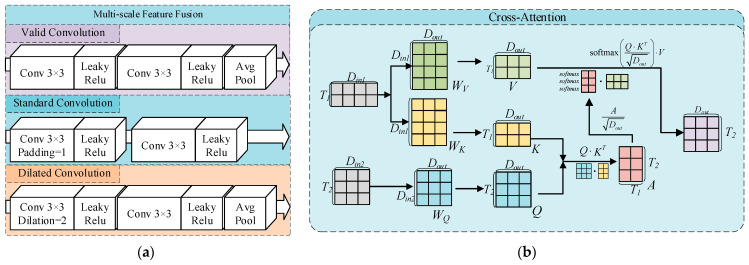
Fusion Strategy: (**a**) Multi-scale Feature Fusion Structure; (**b**) Cross-Attention Feature Fusion Structure.

For the fusion of one-dimensional data, the MFF module is constructed, as illustrated in Figure 3a. Features are extracted at the original, down-sampled, and dilated convolution scales, enabling a comprehensive acquisition of time–frequency representations from one-dimensional signals. For cross-modal feature fusion, as shown in Figure 3b, in this study, we adopted the CA module, since both the one-dimensional sensor signals and the meniscus images reflect the temperature state at the solid–liquid interface. In this process, the meniscus image features are used as the Query to retrieve the most informative features from the past period, thereby generating Key–Value pairs and producing fused features.

The CA network integrates multimodal information by establishing an interaction mechanism between feature representations. Given the main feature $X_{t}\in R^{d\times h_{t}\times w_{t}}$ and the auxiliary fusion feature $X_{p}\in R^{d\times h_{p}\times w_{p}}$, the network first generates the Query, Key, and Value vectors through linear projections:

(3) $$\begin{array}{l} Q=W_{q}X_{t}\\ K=W_{k}X_{p}\\ V=W_{v}X_{p} \end{array}$$

The cross-modal attention weights are then computed by evaluating the Query-Key similarity, followed by the aggregation of the Value vectors:

(4) $$Z=\mathrm{Softmax}\left(\frac{QK^{T}}{\sqrt{d_{k}}}\right)V$$

This structure enables the main feature representation to adaptively focus on the most relevant information contained in the auxiliary modality, thus achieving adaptive cross-modal feature alignment.

### 3.3. Regression Strategy

**Figure 4:**
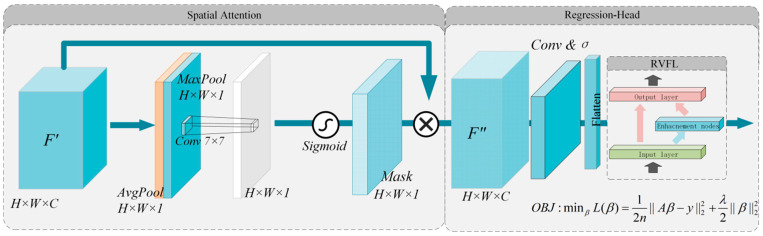
Regression Strategy Based on RVFL.

The regression module is constructed as shown in Figure 4, consisting of an SA module, average pooling compression, a flattening layer, and the RVFLN. The input to the SA module is the feature $F^{\prime \prime }$ with dimensions H×W×C. This feature is subjected to channel-wise average pooling and max pooling, resulting in two H×W×1 spatial descriptors. These descriptors are then concatenated along the channel dimension, followed by a 7×7 convolution and Sigmoid activation to generate the weight coefficient Ms. Finally, $F^{\prime }$ is multiplied element-wise with Ms to obtain the spatially weighted feature $F^{\prime \prime }$, as expressed in Equations (5) and (6).

(5) $$M_{s}=\sigma \left(f^{7\times 7}\left(\left[A_{p}\left(F\right);M_{p}\left(F\right)\right]\right)\right)$$

(6) $$F^{\prime \prime }=M_{s}⊗F^{\prime }$$

Here, $M_{s}$ represents the SA weight coefficient of dimension H×W×1, σ is the Sigmoid activation function, $f_{7\times 7}$ denotes a 7×7 convolution kernel, $A_{p}\left(F\right)$ represents average pooling, $M_{p}\left(F\right)$ represents max pooling, $F^{\prime \prime }$ is the output feature map, ⊗ represents element-wise multiplication, and $F^{\prime }$ is the input feature map.

After the SA mechanism outputs the features, they undergo average pooling compression followed by a flattening operation. The flattening layer converts the multi-dimensional feature map into a one-dimensional vector, which is then fed into the RVFLN for regression modeling. Its core purpose is to enhance feature diversity through fixed random projections, while only the output weights are trainable. During forward propagation, a fixed random layer is generated, and the final output is obtained through feature concatenation and linear prediction, as shown in Equations (7) and (8).

(7) $$H=\sigma \left(XW_{random}+b_{random}\right)$$

(8) $$\overset{\wedge }{y}=\left[X|H\right]\beta =A\beta$$

Here, X represents the input feature, $W_{random}$ and $b_{random}$ denote the randomly generated weight matrix and bias vectors, σ is the activation function, β is the trainable output weight, A is the concatenated feature, and $\overset{\wedge }{y}$ is the predicted output. The optimization objective, given in Equation (9), consists of the mean squared error with L2 regularization. By differentiating the objective function and setting the gradient to 0 in Equation (10), the analytical solution of β can be obtained, as shown in Equation (11).

(9) $$\min_{\beta }L\left(\beta \right)=\frac{1}{2}||A\beta -y|{|}_{2}^{2}+\frac{\lambda }{2}||\beta |{|}_{2}^{2}$$

(10) $$\nabla L\left(\beta \right)=\frac{\partial }{\partial \beta }\left[\frac{1}{2}{\left(A\beta -y\right)}^{T}\left(A\beta -y\right)+\frac{\lambda }{2}\beta^{T}\beta \right]=A^{T}\left(A\beta -y\right)+\lambda \beta$$

(11) $$\beta ={\left(A^{T}A+\lambda I\right)}^{-1}A^{T}y$$

Through this analytical solution, the optimal value of β can be computed in a single step for a given input.

However, in the present task, the input to the RVFLN comprises flattened fused features, which results in extremely large matrices when computing the analytical solution. This leads to prohibitively high memory consumption. To address this issue, an iterative optimization method is adopted to solve for β. The objective function, gradient computation, and parameter update rule for the iterative method are given in Equations (12)–(14).

(12) $$\min_{\beta }L\left(\beta_{t}\right)=\frac{1}{2|B|}||A_{B}\beta_{t}-y_{B}|{|}_{2}^{2}+\frac{\lambda }{2}||\beta_{t}|{|}_{2}^{2}$$

(13) $$\nabla L\left(\beta \right)=\frac{1}{\left|B\right|}A_{B}^{T}\left(A_{B}\beta_{t}-y_{B}\right)+\lambda \beta_{t}$$

(14) $$\beta_{t+1}=\beta_{t}-\eta \nabla L\left(\beta_{t}\right)$$

Here, $B\subset \{1,2,\ldots n\},\left|B\right|<<n$ represents a mini-batch sampled from the full dataset of n training samples.

## 4. Experimental Setup and Results

This chapter will conduct experiments based on the network structure and evaluation indicators presented in the previous section. The algorithm presented in this paper is implemented using the open-source PyTorch deep learning framework (version 2.4.1). Regarding the hardware configuration, the CPU is an Intel (R) Core (TM) i9-9900 @ 3.10GHz, and the GPU is an NVIDIA GeForce RTX 3060. Regarding network hyperparameters, Adam was selected as the global optimizer, the batch size was set to 8, and training was conducted for 30 epochs. The experiments involved in this paper imposed restrictions on the global tensor flow dimensions during tensor computations, the tensor flow between the main components in the network is limited to 128×64×64. During the regression process, the computational load is reduced through downsampling or pooling.

### 4.1. Work Content

Based on the algorithmic strategy proposed in Section 3, this section reports the process of progressively constructing, training, and testing the network model.

#### 4.1.1. Seeding Temperature State Regression Network Based on SA-CA-MFF-RVFLN

**Figure 5:**
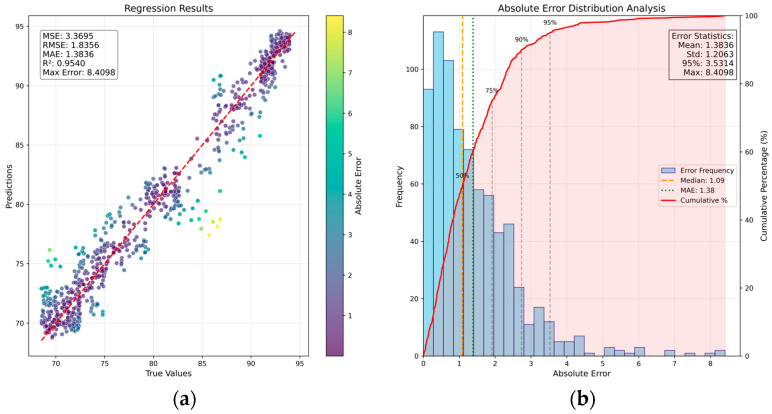
Sample distribution and error distribution based on SA-CA-MFF-RVFLN: (**a**) scatter plot of sample distribution; (**b**) bar chart of sample error distribution.

As shown in Figure 2, the SA-CA-MFF-RVFLN framework comprises four main components: data collection and preprocessing, image feature extraction, fusion strategy, and regression strategy. The latter three modules constitute the end-to-end training portion of the network. Following the training procedure outlined before, the performance of the trained network was evaluated, and the corresponding results are presented in Figure 5. Figure 5a shows the distribution of test samples, and Figure 5b depicts the histogram of prediction errors.

For the detection task, the following evaluation metrics were computed: MSE, RMSE, MAE, R2, MaxError [24], and P95 (error). Among the selected evaluation metrics, P95 (error) denotes the threshold below which 95% of the prediction errors fall, providing insights into the model’s typical performance on the majority of the data. As shown in Figure 5, the proposed SA-CA-MFF-RVFLN achieves the following performance on the test set: MSE = 3.36, RMSE = 1.83, MAE = 1.38, MaxError = 8.40, and P95 (error) = 3.53. The majority of test samples lie close to both sides of the ideal distribution line, demonstrating that the proposed network can accurately capture the mapping relationship between multimodal input features and the target output. Additional experiments are reported in the following subsections to further analyze the effectiveness of the method.

#### 4.1.2. Temperature State Classification Based on Meniscus Images

**Figure 6:**
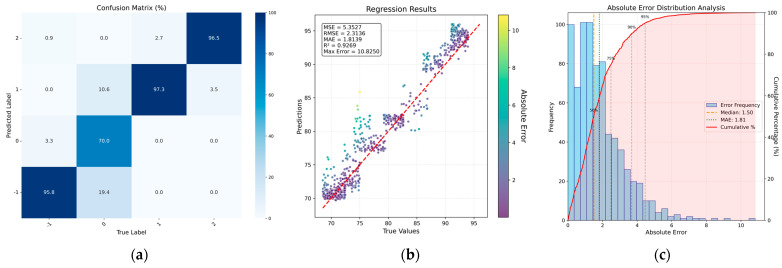
Results of classification and detection based on meniscus images: (**a**) classification result matrix of temperature state; (**b**) scatter plot of sample distribution; (**c**) bar chart of sample error distribution.

As detailed in Section 3.1, to simulate the visual assessment process performed by experienced engineers during production, in this study, we first employed a ResNet-based model to extract meniscus image features and classify temperature intervals. On-site engineers were invited to annotate the dataset, assigning each aligned sample to one of four temperature intervals according to the visual characteristics of the meniscus: low temperature (Low), appropriate temperature (Normal), high temperature (High), and excessively high temperature (Critical). These four intervals were selected because excessively low temperatures may cause crystallization to occur at very low or even zero pulling speeds, potentially leading to large-scale surface crystallization and unsafe conditions. Conversely, at sufficiently high temperatures, the meniscus will melt completely. With the practical objective of temperature control, these extreme cases are unlikely to be misclassified. The classification results are summarized in Figure 6a and Table 1.

For the temperature state classification task, a confusion matrix was plotted on the final test set. In addition, Recall, Precision, F1-score, and Accuracy were computed as evaluation metrics. As observed from Figure 6a and Table 1, variations in the meniscus visual features exhibit clear distinctions across different temperature intervals. These results indicate that temperature state classification can be effectively accomplished using image information alone.

#### 4.1.3. Detection of Temperature Based on Meniscus Images

Based on the principle described in Section 2 regarding the potential relationship between meniscus features and the resulting seeding duration, the classification head in Section 4.1.2 was replaced with an MLP-based regression head for training. Figure 6b,c shows the output results of the network.

As shown in Figure 6b,c, the feasibility of using image-extracted features to detect the seeding temperature is verified. The performance metrics achieved are MSE = 5.35, RMSE = 2.31, MAE = 1.89, MaxError = 10.8, and P95 (error) = 4.48. From the sample distribution plot, it can be observed that when the network relies solely on image features, the predicted values tend to cluster within specific intervals (e.g., corresponding to horizontal axis values 68–73 and 79–83). This phenomenon arises because, in actual Czochralski crystal growth experiments, the search for an appropriate seeding temperature frequently stabilizes within these ranges, leading to a higher proportion of collected samples exhibiting similar meniscus characteristics. Consequently, the extracted image features lack sufficient granularity, limiting their ability to distinguish subtle differences within these dense intervals.

#### 4.1.4. Detection of Temperature States Based on Multi-Sensor Fusion Input

**Figure 7:**
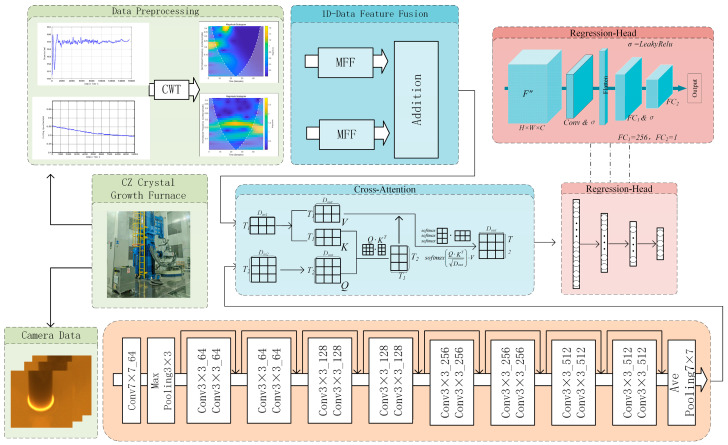
Network structure of CA-MFFRN.

To further enhance the discrimination capability of the model, temperature signals from two pyrometers installed on the Czochralski furnace were incorporated as additional inputs to complement the meniscus image features. A multi-sensor fusion regression network, CA-MFFRN, was constructed, as illustrated in Figure 7.

**Figure 8:**
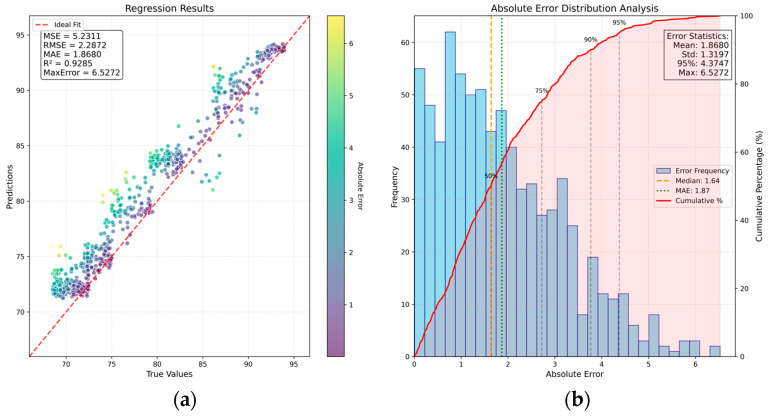
Sample distribution and error distribution based on CA-MFFRN: (**a**) scatter plot of sample distribution; (**b**) bar chart of sample error distribution.

The training results are presented in Figure 8. It can be seen that the CA-MFFRN network achieves MSE = 5.23, RMSE = 2.28, MAE = 1.86, MaxError = 6.52, and P95 (error) = 4.37. Compared with the results in Section 4.1.3, multiple performance metrics show improvements, demonstrating the effectiveness of integrating multi-sensor information. Additionally, the predicted values in different sample regions exhibit a more dispersed distribution, indicating that sensor fusion inputs help mitigate the output-value convergence observed when only image features are used.

**Figure 9:**
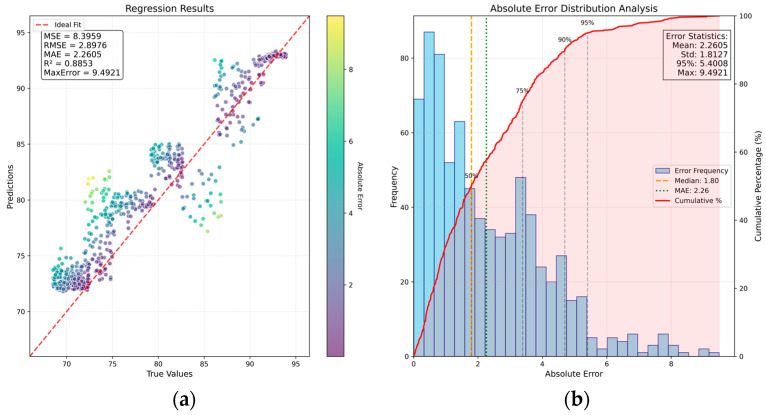
Sample distribution and error distribution based on the concatenation mechanism: (**a**) scatter plot of sample distribution; (**b**) bar chart of sample error distribution.

Next, a fusion mechanism based on concatenation is adopted to replace the CA in the fusion module. The features from multiple sensors are directly concatenated and trained under the same conditions. The test results are shown in Figure 9. According to Figure 8 and Figure 9, simply concatenating the sensor data of different modalities cannot associate the potential cross-modal features between sensors, and there is a risk of interfering with the network. Therefore, using a cross-attention mechanism to fuse cross-modal features has a positive effect.

#### 4.1.5. Seeding Temperature Detection Network Based on SA–CA–MFFRN

**Figure 10:**
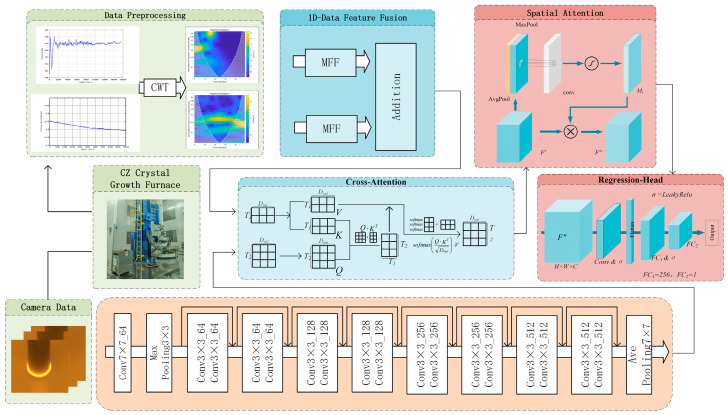
Network structure of SA–CA–MFFRN.

**Figure 11:**
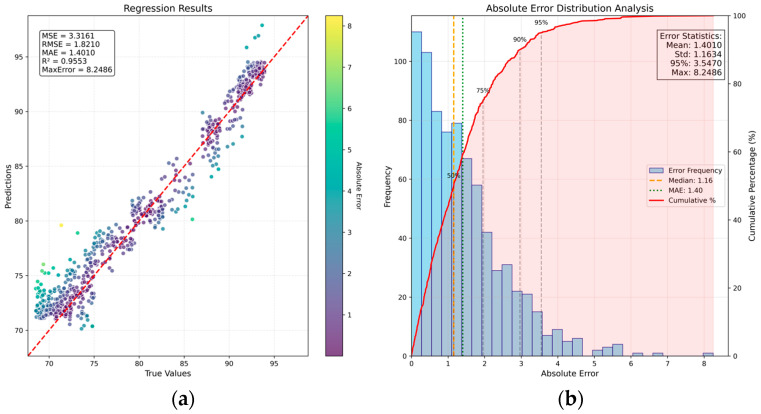
Sample distribution and error distribution based on SA–CA–MFFRN: (**a**) scatter plot of sample distribution; (**b**) bar chart of sample error distribution.

Based on the previous network, further improvements were introduced so that the combined multimodal features could better capture the mapping relationship between the input features and the target output values. To make the network focus more effectively on informative components within the fused features, an SA mechanism was incorporated, as described in Section 4.1.4, to generate attention-based weighting masks. Following the structure illustrated in Figure 10, the SA–CA–MFFRN network was constructed, and training was performed on the same dataset. The corresponding results are shown in Figure 11.

As shown in Figure 11, the SA–CA–MFFRN network achieves MSE = 3.31, RMSE = 1.82, MAE = 1.41, MaxError = 8.24, and P95 (error) = 3.54. After incorporating the SA mechanism, multiple performance indicators show notable improvements, validating the enhanced effectiveness of the multi-sensor feature representation.

### 4.2. Results

**Figure 12:**
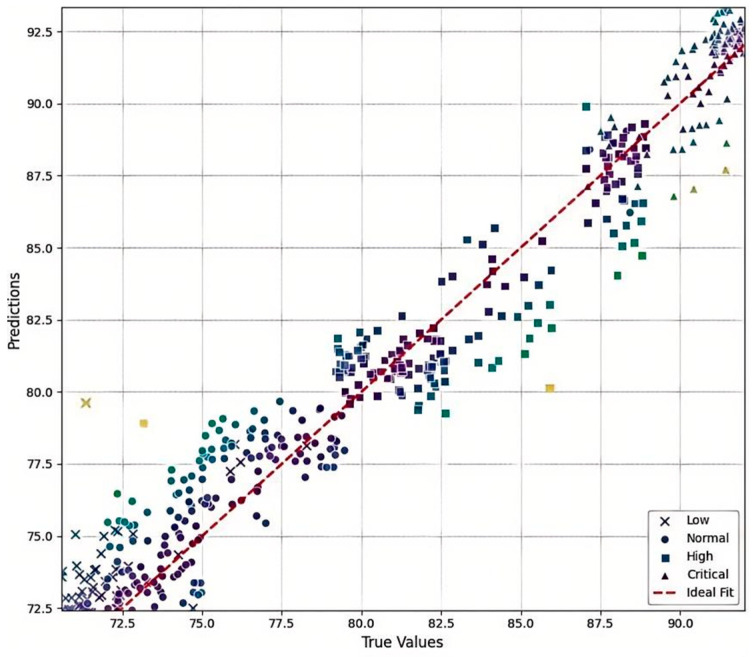
Local distribution map of sample.

Figure 12 presents a local view of the test sample distribution when regression is performed using image information, with the original classification labels superimposed. Different marker styles represent the four temperature state categories. It can be seen that, even under regression, samples belonging to adjacent categories still exhibit distinguishable boundaries, and individual samples cluster around the ideal distribution curve. This indicates that regression provides more refined discrimination than classification, without losing the inherent relationships among features. Regression not only quantifies fine-grained variations within the same category but also produces continuous outputs, which can offer more informative guidance for subsequent temperature control decisions.

Combining Table 2 with Figure 6b,c and Figure 8, we can see that effectively fusing one-dimensional sensor signals with image features leads to improved performance metrics. Moreover, the predicted values within previously convergent regions become more dispersed, mitigating the output-value convergence observed when only image features serve as inputs.

Furthermore, by combining Table 2 with Figure 8 and Figure 11, we can see that through SA-based weighted masking of the fused features, most performance indicators on the test set further improve, and the convergence phenomenon among predicted sample values is further mitigated. These results indicate that the introduced SA mechanism positively contributes to the current prediction task.

Finally, in the context of this study, the relationship between the input sensor information and the output seeding duration is approximately linear. The improved method, SA–CA–MFF–RVFLN, includes RVFLN in place of the traditional MLP regression, thereby reducing potential overfitting risks associated with MLP’s strong nonlinear fitting capability and lowering the total number of model parameters. In Section 4.1.1, the average inference time for a single sample is 0.089 s, whereas in Section 4.1.5, the average inference time is 0.111 s. By jointly examining Table 2 and Figure 5 and Figure 11, we can see that the proposed method achieves faster offline inference while maintaining comparable prediction performance. Moreover, the improved network in Section 4.1.1 and the network in Section 4.1.5 share the same backbone structure, and both achieve similar performance results, validating the effectiveness of the backbone’s feature extraction and fusion design.

However, our experiments still have several limitations. Due to the actual operating conditions of the Czochralski furnace, certain temperature intervals occur only rarely, resulting in a limited number of sample points within these ranges (e.g., target values around 82–87). Under identical training conditions, the model’s feature learning in these sparse intervals is relatively inadequate, which is reflected by reduced prediction accuracy in these regions. Since image features vary continuously with temperature, in future work, researchers may consider introducing mechanisms related to meniscus evolution or prior temperature knowledge to compensate for the scarcity of samples in such intervals.

Overall, in this study, we successfully transformed the task of detecting the seeding temperature from a conventional classification problem into a regression problem by using the seeding duration as the target variable. This formulation preserves the continuity of the underlying temperature field and provides quantitative guidance for subsequent temperature control operations. Moreover, the results demonstrate the positive impact of introducing one-dimensional furnace sensor data, which enhances image-based monitoring of the thermal environment during the seeding stage of crystal growth.

## 5. Conclusions

To address the current challenge of the inability to directly detect the interface temperature at the beginning of the seeding stage in Czochralski silicon monocrystal growth, this paper proposes a multimodal fusion network-based detection method. By fusing information collected from sensors at different positions in the crystal growth furnace, as well as the seeding duration, this method allows us to establish an indirect relationship between the temperature (which cannot be directly measured) and the sensor data using a Random Vector Functional Link Network (RVFLN), thereby re-quantifying the temperature. The proposed SA–CA–MFF–RVFLN model effectively extracts representative features from the time-frequency domain signals of the sensors and the meniscus images and learns the mapping relationship from the features to the quantified values, achieving quantitative temperature measurement. However, two critical challenges remain, informing future focus areas. Firstly, in practical industrial settings, collecting sensor data under various temperature conditions often requires lengthy experimental cycles. Therefore, network training with small sample sizes or partially missing data still faces bottlenecks in achieving stable and reliable predictions. Secondly, there is still room for further research on the more effective utilization of sensor information in this regard. For instance, multiple axial sensors and radial temperature sensors can be added to the single crystal furnace equipment to take advantage of the temperature gradient variations in both the axial and radial directions. Addressing these issues could have long-term significance in advancing the intelligent development of crystal growth technologies.

## Figures and Tables

**Table 1:** Performance indicators of ResNet network classification results.

	Accuracy	Precision	Recall	F1-Score
Critical	0.90	0.96	0.96	0.95
High		0.87	0.97	0.92
Normal		0.95	0.70	0.81
Low		0.85	0.96	0.90

**Table 2:** Comparison of regression models’ performance metrics.

	MSE	RMSE	MAE	MaxError	P95 (Error)
ResNet-MLP	5.35	2.31	1.89	10.80	4.48
CA-MFFRN	5.23	2.28	1.86	6.52	4.37
SA–CA–MFFRN	3.31	1.82	1.41	8.24	3.54
SA-CA-MFF-RVFLN	3.36	1.83	1.38	8.40	3.54

## Data Availability

The data presented in this study are available on request from the corresponding author. The data are not publicly available due to legal and privacy considerations.
